# Knocking down mitochondrial iron transporter (MIT) reprograms primary and secondary metabolism in rice plants

**DOI:** 10.1093/jxb/erv531

**Published:** 2015-12-17

**Authors:** Gianpiero Vigani, Khurram Bashir, Yasuhiro Ishimaru, Martin Lehmann, Fabio Marco Casiraghi, Hiromi Nakanishi, Motoaki Seki, Peter Geigenberger, Graziano Zocchi, Naoko K. Nishizawa

**Affiliations:** ^1^Dipartimento di Scienze Agrarie e Ambientali-Produzione, Territorio, Agroenergia, Università degli Studi di Milano, via Celoria 2-20133Milano, Italy; ^2^RIKEN Center for Sustainable Resource Science, 1-7-22 Suehiro-cho, Tsurumi-ku, , Yokohama, Kanagawa 230-0045, Japan; ^3^Graduate School of Science, Tohoku University, 6-3, Aramaki-aza Aoba, Aoba-ku, Sendai 980-8578, Japan; ^4^Plant Molecular Biology (Botany) and Plant Metabolism, Department Biology I, Ludwig-Maximilians-Universität München (LMU), Großhaderner Straße 2, D-82152 Planegg-Martinsried, Germany; ^5^Department of Global Agricultural Sciences, Graduate School of Agricultural and Life Sciences, The University of Tokyo,Tokyo, Japan; ^6^CREST, JST, 4-1-8 Honcho, Kawaguchi, Saitama 332-0012, Japan; ^7^Research Institute for Bioresources and Biotechnology, Ishikawa Prefectural University, 1-308 Suematsu, Nonoichi-shi, Ishikawa 921-8836, Japan

**Keywords:** Iron, iron deficiency, metabolomics, mitochondria, Oryza sativa, transcriptomics

## Abstract

Iron (Fe) is an essential micronutrient for plant growth and development, and its reduced bioavailability strongly impairs mitochondrial functionality. In this work, the metabolic adjustment in the rice (*Oryza sativa*) mitochondrial Fe transporter knockdown mutant (*mit-2*) was analysed. Biochemical characterization of purified mitochondria from rice roots showed alteration in the respiratory chain of *mit-2* compared with wild-type (WT) plants. In particular, proteins belonging to the type II alternative NAD(P)H dehydrogenases accumulated strongly in *mit-2* plants, indicating that alternative pathways were activated to keep the respiratory chain working. Additionally, large-scale changes in the transcriptome and metabolome were observed in *mit-2* rice plants. In particular, a strong alteration (up-/down-regulation) in the expression of genes encoding enzymes of both primary and secondary metabolism was found in mutant plants. This was reflected by changes in the metabolic profiles in both roots and shoots of *mit-2* plants. Significant alterations in the levels of amino acids belonging to the aspartic acid-related pathways (aspartic acid, lysine, and threonine in roots, and aspartic acid and ornithine in shoots) were found that are strictly connected to the Krebs cycle. Furthermore, some metabolites (e.g. pyruvic acid, fumaric acid, ornithine, and oligosaccharides of the raffinose family) accumulated only in the shoot of *mit-2* plants, indicating possible hypoxic responses. These findings suggest that the induction of local Fe deficiency in the mitochondrial compartment of *mit-2* plants differentially affects the transcript as well as the metabolic profiles in root and shoot tissues.

## Highlight

Knocking down mitochondrial iron transporter decreases respiratory chain activity in *mit-2* rice mutants, accompanied by comprehensive changes in the transcriptome and metabolome, with these responses being differentially regulated in roots and shoots.

## Introduction

Iron (Fe) is an essential element for plants, as it is part of the prosthetic group of different proteins directly involved in photosynthesis and respiration (haem or Fe–sulphur [Fe–S] clusters), and participates in other key metabolic pathways, such as nitrogen assimilation and scavenging of reactive oxygen species (ROS) (Couturier *et al.*, 2013; Balk and Schaedler, 2014).

Despite its abundance in soils, Fe is scarcely soluble, especially under alkaline and aerobic conditions (Guerinot and Ying, 1994). Plants growing under low Fe availability, such as in calcareous soils, often suffer from Fe deficiency, which reduces growth, crop yield, and quality (Marschner, 1995). The molecular mechanisms of Fe uptake from the rhizosphere have been extensively studied, and two strategies have been identified in the plant kingdom: Strategy I, the reduction strategy; and Strategy II, the chelation strategy (Bashir *et al.*, 2010, 2013a; Kobayashi and Nishizawa, 2012). Dicots and non-graminaceous monocots utilize Strategy I, whereas graminaceous plants utilize Strategy II and possess a specific ability to synthesize the so-called phytosiderophores, Fe(III) chelators belonging to the mugineic acid family (Marschner and Romheld, 1994). These strategies have been previously considered mutually exclusive, but some exceptions were recently reported in which Strategy II plants possess partial Strategy I uptake systems (Bughio *et al.*, 2002; Bashir *et al.*, 2011b, 2013a; Ishimaru *et al.*, 2011; Kobayashi and Nishizawa, 2012).

Despite the wealth of knowledge that has been gained concerning the processes by which plants can respond to Fe deficiency, the mechanisms of Fe sensing and signalling are not yet fully understood. It has been recently reviewed that some transcription factors involved in the Fe deficiency-induced responses might play a role as Fe sensors in the cell, while several molecules might be good candidates as Fe signals (Kobayashi and Nishizawa, 2014). At the cellular level, the regulation of Fe deficiency-mediated responses in plants is a complex mechanism that requires the orchestration of all compartments. It has been suggested that cellular organelles such as mitochondria might regulate Fe deficiency-induced responses through retrograde signalling pathways that are still poorly known in plants (Vigani *et al.*, 2013a, b). Plant mitochondria are central hubs in energy conversion and redox homeostasis, and are connected to metabolic pathways residing in different subcellular compartments. Hence, mitochondria are ideally placed to act as sensors of the energetic and metabolic status of the cell (Sweetlove *et al.*, 2007; Millar *et al.*, 2011). Perturbations of the cellular energy status can lead to a re-configuration of mitochondrial activities, which in turn have profound effects on other cellular compartments, including major changes in the nuclear gene expression (NGE) and photosynthetic activity (Schwarzländer *et al.*, 2012).

In rice plants, a mitochondrial iron transporter (MIT) has recently been identified (Bashir *et al.*, 2011c). The MIT protein seems to act as a high-affinity Fe uptake system in plant mitochondria in analogy with the yeast MRS3/4 homologous transporters, that are thought to serve as high-affinity ferrous ion transporters which are essential in the absence of other low-affinity mitochondrial Fe transporters (Froschauer *et al.*, 2009). In rice, *mit* is an essential gene, with *mit* knockdown mutants (*mit-2*) exhibiting a slow growth phenotype and a reduced chlorophyll content. In *mit-2* mutants, T-DNA is integrated 604bp upstream of the ATG codon and the expression of MIT is ~30% less compared with WT plants (Bashir *et al.*, 2011c). *mit-2* exhibits a significant reduction in root and shoot dry weight as well as in the root and shoot length, leaf width, and chlorophyll content compared with WT plants (Bashir *et al.*, 2011c). Moreover, the *mit-2* mutation significantly alters the cellular Fe homeostasis and localization (Bashir *et al.*, 2013b). Indeed, in *mit-2* plants, the mitochondrial Fe concentration is low while the total Fe concentration is high compared with WT plants (Bashir *et al.*, 2011c). Besides the mitochondrial Fe transport, the *mit-2* mutation affects Fe–S cluster assembly, in agreement with previous observations in other organisms. In yeast and mammals, the loss of mitochondrial Fe transport affects haem and Fe–S cluster synthesis (Zhang *et al.*, 2005, 2006; Shaw *et al.*, 2006). In rice, the knock down of MIT results in a decrease in total and mitochondrial aconitase activity; therefore, it has been suggested that Fe–S cluster synthesis might be affected at both the mitochondrial and cytosolic levels.

In this work, we have characterized the effect of mitochondrial impairments of Fe transport on the overall metabolism of the cell, using *MIT* knocked-down mutant rice plants (Bashir *et al.*, 2011a, c). Combined transcriptomics and GC-MS-based metabolomics approaches were used to provide evidence that specific Fe deficiency localized in mitochondria in response to a knockdown mutation of MIT leads to global changes in metabolism.

## Materials and methods

### Plant growing conditions

Rice seeds (*Oryza sativa* L. cv. Dongjing) of the WT and *mit-2* were germinated for 1 week on paper towels soaked with distilled water at room temperature. After 1 week, seedlings were transferred to a nutrient solution with the following composition: 0.7mM K_2_SO_4_, 0.1mM KCl, 0.1mM KH_2_PO_4_, 2.0mM Ca(NO_3_)_2_, 0.5mM MgSO_4_, 10 μM H_3_BO_3_, 0.5 μM MnSO_4_, 0.2 μM CuSO_4_, 0.5 μM ZnSO_4_, 0.05 μM Na_2_MoO_4_, and 100 μM Fe-EDTA, as described previously (Suzuki *et al.*, 2006), and grown for 3 weeks. Plants were grown with a day/night regime of 16/8h and 25 °C/20 °C, and a photosynthetic photon flux density (PPFD) of 200 μmol photons m^−2^ s^−1^ at the plant level. The pH of the nutrient solution was adjusted daily to 5.5 with 1M HCl. The solution was renewed every 5 d. Plants were harvested at noon.

### Root oxygen consumption rate, mitochondrial purification, and western blot analyses

The oxygen consumption rate of rice root tips was measured as described previously (Vigani *et al.*, 2009). To determine the contribution of mitochondrial respiration to the O_2_ consumption rate of root tissues, specific inhibitors of the respiratory chain were used (O_2_ consumption rate on three independent biological replicates, *n*=3) (Vigani *et al.*, 2009).

Mitochondria were isolated from rice roots as described previously (Vigani *et al.*, 2009). Roots of 3-week-old rice plants were homogenized with a mortar and pestle in 0.4 M mannitol, 25 mM MOPS, pH 7.8, 1 mM EGTA, 8 mM cysteine, and 0.1% (w/v) bovine serum albumin (BSA). Cell debris was pelleted by a brief centrifugation at 4000 *g*. The supernatant was re-centrifuged at 12 000 *g* for 15min to pellet mitochondria. The crude mitochondrial pellet was resuspended in 0.4 M mannitol, 10 mM Tricine, pH 7.2, 1 mM EGTA (resuspension buffer, RB) and lightly homogenized with a potter, and mitochondria were purified on a 40, 28, and 13.5% (v/v) percoll (Pharmacia, Uppsala, Sweden) step gradient in RB. The buff-coloured fraction (purified mitochondria) at the interface between 28% and 40% percoll was collected and washed by differential centrifugation in RB. The purified mitochondria were frozen and stored at –80 °C until use. The Fe content in purified mitochondria was determined by inductively coupled plasma (ICP)-MS spectroscopy (Varian, Fort Collins, CO, USA) after mineralization in HNO_3_ at 100–120 °C as described previously (Vigani *et al.*, 2009) (the mitochondrial fraction was purified and the Fe content was determined on three independent biological replications, *n*=3).

Mitochondrial proteins were loaded on a discontinuous SDS–polyacrylamide gel and processed as described previously (Vigani *et al.*, 2009). Western blot analysis was performed as described previously (Vigani *et al.*, 2009), using six different antibodies, corresponding to: NAD9 polyclonal antibody from wheat; NDB1 and NDA1 polyclonal antibodies from potato (Svensson and Rasmusson, 2001); Rieske (polyclonal antibody) from yeast (Balk and Leaver, 2001); the alternative oxidase (AOX); and the porin maize proteins (monoclonal antibodies). Porin antibodies were used as loading control. Mitochondrial purification and western blot analysis were conducted independently on three independent biological replications (*n*=3).

### Analysis of microarray data

The microarray data have already been described briefly (Bashir *et al.*, 2011c). For microarray analysis, samples from three biological replications were pooled together to extract RNA. Microarray analyses were performed with two technical replications. Spots with a *P*-value <0.01, and a ratio >2 in both the Cy3 and Cy5 channels, were considered to be significantly up-regulated. Real-time PCR analysis of selected genes was performed to validate the microarray analysis. We reanalysed the data with a particular focus on genes related to metabolism. For MapMan analysis, the average log_2_ value was calculated for individual annotations in roots and shoots. This log_2_ value was then used to compare the transcriptomic changes in metabolism-related genes using MapMan 3.6.0RC1 (Kim *et al.*, 2012).

### Metabolomic analysis of root and leaf tissues of *mit-2* and WT plants

Metabolites for GC-TOF-MS were extracted using a modified method described in Roessner *et al.* (2001) and Lisec *et al.* (2006). Leaf and root tissues were frozen and homogenized in liquid nitrogen. For extraction, 50mg of ground material was mixed with methanol containing ribitol and C_13_-sorbitol as internal standards. After mixing and incubating at 70 °C, water and chloroform were added to force a phase separation by centrifugation. Only the upper polar phase was dried in a vacuum and used for further analysis. The pellet was derivatized using methoxyaminehydrochloride (20mg ml^−1^ in pyridine) for methoxyamination, and *N*,*O*-Bis(trimethylsilyl)trifluoroacetamide (BSTFA) for silylation. To perform a retention time alignment later on, a mixture of alkanes (C10, C12, C15, C19, C22, C28, and C32) was added to the derivatization mix.

Metabolites were analysed using a GC-TOF-MS system (Pegasus HT, Leco, St Joseph, USA). Baseline correction was done by ChromaTOF software (Leco). For peak alignment and peak annotation, the TagFinder software tool (MPIMP Golm; Luedemann *et al.*, 2008) was used in combination with the Golm Metabolome Database (GMD; Kopka *et al.*, 2005). The metabolites were normalized using the internal standard and the fresh weight.

### Statistical analysis

Student’s *t*-tests were performed using the algorithm embedded in Microsoft Excel (Microsoft, http://www.microsoft.com). The term significant is used in the text only when the change in question has been confirmed to be significant (*P*<0.05) by Student’s *t*-test. All experiments were conducted independently at least three times. For metabolomics, statistical analysis was performed using Excel and the Multi Experiment Viewer (MEV). Data for the HCL tree are log_2_ transformed (Supplementary Fig. S4 at *JXB* online). Principal component analysis (PCA) was performed using the MetaGeneAlyse platform (metagenealyse.mpimp-golm.mpg.de; Daub *et al.*, 2003). Data for the PCA were median centred and log_10_ transformed (metabolomics experiments were conducted using five independent biological replicates, *n*=5).

## Results

### Knocking down the *MIT* gene affects mitochondrial functionality in rice plant roots

The partial loss of function of *MIT* (*MIT* expression was 30% lower in *mit-2* than in WT plants as reported by Bashir *et al.*, 2011c) affects mitochondrial functionality in root tissues.

**Fig. 1.**
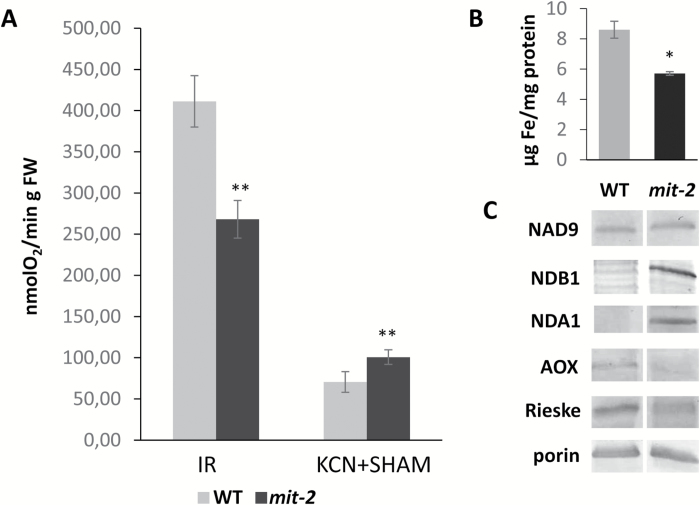
Biochemical characterization of purified mitochondria from roots of *mit-2* and WT plants showed alterations in the respiratory chain in *mit-2*. (A) O_2_ consumption of root tips. The specific inhibitors of the respiratory chain (KCN for cytochrome *c* oxidase and SHAM for alternative oxidase) were added after recording the initial O_2_ consumption rate (IR). The difference between IR and the O_2_ consumption recorded after addition of KCN+SHAM provides the contribution of the mitochondrial O_2_ consumption rate. (B) Mitochondrial Fe concentration in roots. (C) Western blot analysis of *mit-2* and WT roots. For each sample, 10 µg of protein was used. The antibodies used were for: NAD9 (a subunit of complex I), alternative NAD(P)H dehydrogenases (NDB1 and NDA1), AOX (alternative oxidase), Rieske (a subunit of complex III), and porin (used as loading control). Error bars represent the SD. Column bars followed by an asterisk are significantly different from the WT according to Student’s *t*-test (*n*=3, *P<0.01; **P<0.001).

The *in vivo* O_2_ consumption rate (initial rate; IR), determined on root tips, was significantly lower in *mit-2* compared with WT plants (Fig. 1A). By using inhibitors of respiratory chain activity [KCN, a specific inhibitor of complex IV activity; and salycilhydroxamic acid (SHAM), a specific inhibitor of AOX], the contribution of mitochondrial respiration to the total O_2_ consumption by the tissue was also found to be significantly reduced in *mit-2* compared with WT plants (Fig. 1A). The mitochondrial Fe content was measured again for the present study which confirmed that the *mit-2* plants accumulated significantly less Fe in mitochondria compared with WT plants (Fig. 1B). Accordingly, some proteins belonging to the electron transport chain were affected in *mit-2* compared with WT plants (Fig. 1C): NDB1s [external alterative NAD(P) dehydrogenases] and NDA1s [internal alternative NAD(P) dehydrogenases] strongly accumulated in *mit-2* roots compared with WT plants; Fe-containing proteins such as Rieske and AOX decreased in *mit-2* plants (Fig. 1C).

### Transcriptional changes in *mit-2* roots

**Fig. 2.**
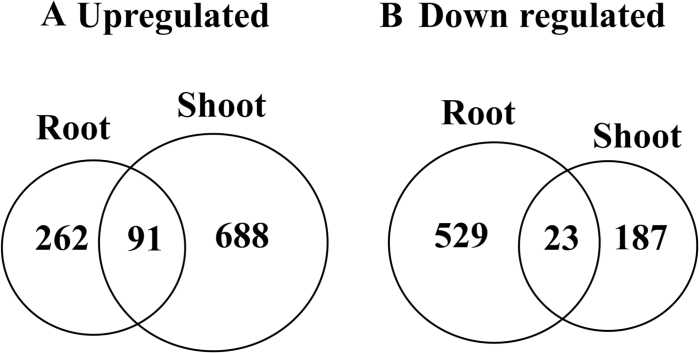
Summary of transcriptomic changes in *mit-2* plants grown under control conditions. (A) Numbers of up-regulated genes identified in root and shoot tissues in *mit-2* with respect to WT plants. (B) Numbers of down-regulated genes identified in root and shoot tissues in *mit-2* with respect to WT plants

**Fig. 3.**
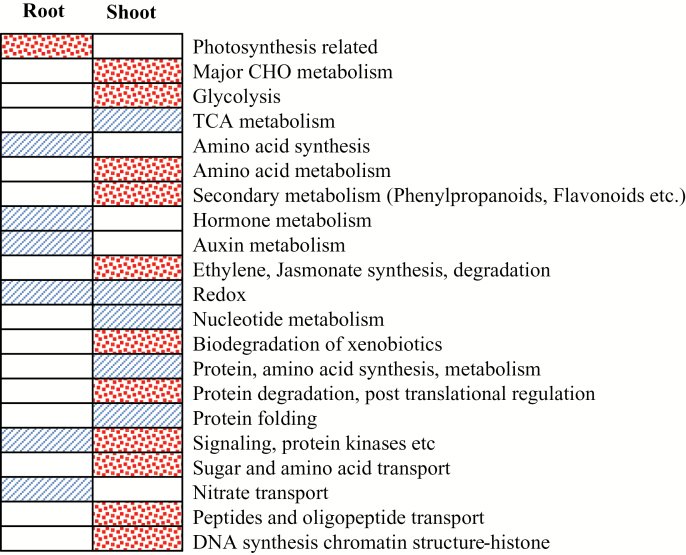
Pageman analysis reflecting changing in *mit-2* root and shoot. The bins shown as stippled boxes are significantly up-regulated, while the bins shown as cross-hatched boxes are significantly down-regulated according to Pageman analysis (Wilcoxon test with BH correction, MapMan 3.6.0RC1). (This figure is available in colour at *JXB* online.)

Real-time PCR analysis confirmed that the expression of *Polygalaturonase isozyme 1, vacuolar iron transporter 2* (*OsVIT2*), endoplasmic reticulum (ER)-type calcium ATPase subfamily member *OsECA1*, and *OsFerroportin 1* was up-regulated in root tissue (Supplementary Fig. S1). These data are in line with the microarray analysis; however, in the microarray analysis, the expression of *OsFerroportin 1* was only slightly up-regulated (1.1 times) in the *mit-2* mutant, thus it is not included in Supplementary Table S1. We reanalysed the microarray data to identify genes related to metabolic changes in *mit-2* roots. The expression of 353 genes was significantly up-regulated in *mit-2* roots compared with WT roots and, among these, the expression of 91 genes was also up-regulated in the shoots (Fig. 2; Supplementary Table S1). On the other hand, the expression of 552 root genes was significantly down-regulated (Fig. 2; Supplementary Table S2). We particularly focused our attention on changes of genes related to metabolism. MapMan analysis clearly indicated that metabolism is significantly reprogrammed in *mit-2* roots compared with those of the WT (Fig. 3; Supplementary Fig. S2). The expression of genes belonging to primary mitochondrial metabolism was affected in *mit-2* plants. The expression of *Os09g0508900* (a member of the mitochondrial substrate transporter family) was significantly up-regulated (Supplementary Table S1), while the expression of *Os01g0226600* and *Os02g0530100* (C4-dicarboxylate transporter/malic acid transport protein family proteins) was down-regulated in roots (Supplementary Table S2). The expression of this gene is reported to be regulated by an excess of Fe in the shoot (Bashir *et al.*, 2014). Furthermore, genes related to the tricarboxylic acid (TCA) cycle, such as phospho*enol*pyruvate carboxykinase 1 (PEPCK1; *Os10g0204400*), showed a significant down-regulation in *mit-2* plants, as did the gene encoding a pyruvic acid orthophosphate dikinase (PPDK) (Supplementary Table S2). The expression of two pyruvic acid orthophosphate dikinase genes (*Os05g0405000* and *Os03g0432100*) was also significantly down-regulated in *mit-2* roots. A strong induction of fructose-2,6-bisphosphatase genes (*Os08g0367300*, *Os07g0212400*, and *Os02g012900*) was observed in *mit-2* roots and shoots, while other glycolytic genes showed a significant down-regulation (Supplementary Table S2).

In general, several genes related to the whole metabolism were significantly down-regulated in roots. The down-regulated genes are mainly grouped in the phenylpropanoid and flavonoid pathways, while the up-regulated genes belong to the pathways of terpene biosynthesis (Supplementary Tables S1, S2).

### Transcriptional changes in *mit-2* shoots

In shoots, the expression of 779 genes was significantly up-regulated in *mit-2* plants compared with WT plants, while the expression of 210 genes was significantly down-regulated (Fig. 2; Supplementary Tables S3, S4). Real-time PCR analysis confirmed the microarray data. The expression of *Polygalaturonase isozyme 1*, *OsVIT2*, *OsECA1*, and *OsFerroportin 1* was significantly up-regulated in shoots tissue (Supplementary Fig. S3). MapMan analysis clearly indicated that metabolism changed significantly in *mit-2* shoots compared with those of the WT (Fig. 3; Supplementary Fig. S3). Genes related to major CHO metabolism, glycolysis, amino acid metabolism, ethylene and jasmonate synthesis and degradation, sugar and amino acid transport, as well as peptide and sugar transport were significantly up-regulated in *mit-2* plants compared with WT plants (Fig. 3). As observed in roots, few genes encoding proteins participating in mitochondrial metabolism were differentially expressed in *mit-2* plants compared with WT plants. Five genes were up-regulated and two of them encode the alternative pathways of the respiratory chain (*Os08g0141400*, NDB3, putatively related to the internal ND II type; *Os04g0600300*, AOX1B) (Supplementary Table S3). *Os05g0331200*, encoding external type II alternative NDs, was down-regulated (Supplementary Table S4). Additionally, two S-type anion channel genes related to mitochondrial transport were up-regulated: *Os01g0385400*, SLAH3; and *Os01g0623200*, SLAH2 (Supplementary Table S3). These genes are homologues to SLAC1 which is required for plant guard cell S-type anion channel function in stomatal signalling (Vahisalu *et al.*, 2008). Three genes encoding proteins of the TCA pathway were up-regulated in *mit-2* plants (*Os05g0405000*, PPDK; *Os07g0529000*, isocitrate lyase, ICL; *Os01g0743500*, NADPH malic enzyme3) (Supplementary Table S3), while the expression of two genes was down-regulated (*Os10g0204400*, PCK1; *Os01g0829800*, malate dehydrogenase, MDL) (Supplementary Table S4).

Similarly to the changes observed in roots, genes encoding fructose-2,6-bisphosphatase (*Os08g0367300*, *Os07g0212400*, and *Os02g012900*) were up-regulated in *mit-2* shoots (Supplementary Table S3) together with the gene encoding HEXOKINASE1 (HXK, *Os01g0722700*), while other glycolytic genes showed a significant down-regulation (Supplementary Table S4).

Expression of 53 genes belonging to secondary metabolism was significantly affected in *mit-2* shoots. In contrast to what was observed in roots, the majority (85%) of differentially expressed genes were up-regulated (Fig. 3; Supplementary Table S3 . The up-regulated genes are mainly grouped in the terpenoid, phenylpropanoid, and flavonoid pathways, suggesting a strong induction of such pathways in the mutant when compared with WT plants. The down-regulated genes were: *Os10g0108700* and *Os07g0179300* (transferase family proteins), *Os10g0118000* (*o*-methyltransferase), *Os11g0708100* (laccase7), *Os07g0526400* (narigerin-chalcone synthase), *Os02g713900* (HMGR2, 3-hydroxy-3-methylgluraryl-CoA reductase 2), *Os10g0533500* (beta-hydroxylase 1), and *Os07g0179300* (2 methyl-6-phytyl-1,4-benzoquinone) (Supplementary Table S4).

### Metabolomic changes in *mit-2* plants

**Fig. 4.**
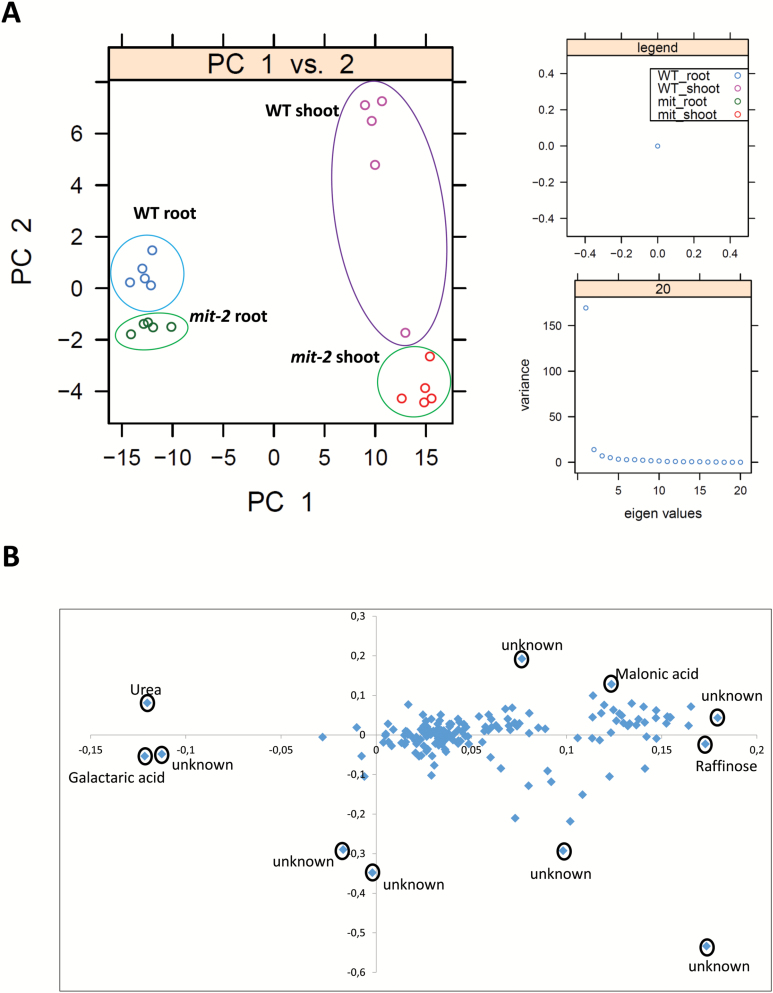
Principal component analysis (PCA) of the metabolomic data set of root and leaf tissues of *mit-2* and WT plants. (A) PCA of metabolite data of five independent biological replicates. Data are median centred and log_10_ transformed. All 159 metabolites were used to perform the analysis. PC1, first principal component; PC2, second principal component. (B) Corresponding loading plot of all metabolites characterized by metabolomic analysis. Metabolites responsible for the PCA separation of the analysis are named in the graph. (This figure is available in colour at *JXB* online.)

To investigate further the role of MIT in the reprogramming of global metabolism, we performed a GC-MS-based metabolomic analysis in both root and shoot tissues of *mit-2* and WT rice plants (Table 1; Fig. 4A, B; Supplementary Fig. S4; Supplementary Table S5). A total of 159 metabolites were detected by GS-TOF-MS analysis (Supplementary Table S5) and 68 of them were identified (Table 1; Supplementary Table S5). Among the identified metabolites (68), 31 (46%) significantly changed their amount in *mit-2* compared with the WT tissues. To obtain a general overview of the metabolic changes, a PCA analysis was performed. The major variance of the metabolomic data was between the different tissues analysed (root and shoot) as explained by PC1. Urea and galactaric acid showed higher amounts in roots than in shoots (galactaric acid was not detected in shoots), while malonic acid and raffinose mainly accumulated in shoots. The separation of the genotypes contributed to the variance along PC2 (Fig. 4A). Thereby the mutant plants are characterized by a higher content of an unknown metabolite (probably sugar) and a lower amount of urea (not detected in the mutant shoots) and malonic acid (not detected in the mutant roots). Interestingly, the separation of the genotypes is stronger in shoots than in roots (Fig. 4B).

**Table 1.** Content of identified metabolites in roots and shoot tissues of *mit-2* and WT rice plants Metabolite changes are expressed as fold changes (*mit-2*/WT ratio).

metabolites	Root		Shoot	
	*mit-2*/WT ratio	*P*-value	*mit-2*/WT ratio	*P*-value
Amino acids (and related compounds)				
Alanine	0.321*	0.0002	1.053	0.9121
Alanineamide	0.909*	0.045	1.144	0.7214
Arginine	0.444	0.3260	1.409	0.1595
Aspartic acid	0.695*	0.0114	0.527*	0.0346
Butanoic acid, 2-amino-	0.010	0.0876	1.396	0.3535
diethanolamine	0.968	0.3535	0.963	0.8709
ethanolamine	1.340	0.9743	1.752*	0.0185
Glutamic acid	1.120*	0.0185	0.763	0.1481
Glutamine	1.000	0.0701	15.655	0.1516
Glycine	0.479*	0.0065	0.763*	0.0065
Guanidine	0.447*	0.0065	2.125*	0.0065
Homoserine	0.414*	0.0008	2.618	0.0629
Isoleucine	0.845	0.0807	1.193	0.5858
Leucine	1.423	0.1310	1.464	*0.4236*
Leucine, cyclo	0.322*	0.0023	0.644	0.5934
Lysine	0.667*	0.0208	0.793	0.5589
Ornithine	68.746	0.2300	4.418*	0.0155
Octopamine	1.302	0.3048	0.690	0.1960
Phenylalanine	0.702*	0.0088	2.125	0.0661
Pyroglutamic acid	0.660*	0.0073	1.493*	0.0073
Proline	0.529	0.0903	1.427	0.4096
Serine	0.906*	0.0441	1.172	0.6675
Serine, *O*-acetyl	0.823	0.0717	2.156	0.1682
Threonine	0.458*	0.0013	1.626*	0.0013
Tryptamine, 5-hydroxy-	1.458	0.3215	3.097	0.1042
Tyrosine	1.129	0.2055	0.743	0.5265
Uracil	1.741	0.7358	1.207	0.7349
Urea	0.472	0.0854	0.000	0.3466
Valine	0.656	0.1226	1.228	0.3969
Carbohydrates (and related compounds)				
Glucose	0.921*	0.0446	1.081	0.8886
Glucose-6-phosphate	2.074	0.4729	1.909*	0.0436
Fructose	0.495*	0.0063	0.487	0.4485
Fructose-1-phosphate	1.000	0.0825	1.655	0.0752
Sucrose	0.563*	0.0034	0.837	0.5371
Raffinose	1.000	0.1999	4.228*	0.0066
Rhamnose	0.555	0.0901	1.583	0.1555
Ribonic acid	1.315	0.4038	2.974*	0.0227
Ribose	0.713	0.0749	1.227	0.6517
Mannose	1.123	0.1411	4.436*	0.0079
Arabinose	0.978	0.0851	1.313	0.3126
Melezitose	1.000	0.0610	1.436	0.0981
Fucose	1.417*	0.0331	1.290	0.3600
Galactaric acid	1.113	0.3409	1	-
Galactinol	0.676	0.9945	1.466	0.1781
Galactosamine, *N*-acetyl-	1.014	0.0755	0.813	0.2831
Galactose	0.704*	0.0248	2.247*	0.0078
Glucoheptonic acid-1,4-lactone	1.091	0.0698	0.726	0.1776
Beta-galactopyranosyl-1,3-arabinoseD	1.000	0.4677	1.525	0.1660
Organic acids (and related compounds)				
Citric acid	0.309*	0.0035	0.740	0.2464
Fumaric acid	1.022	0.0906	6.663*	0.0250
Glutaric acid, 2-oxo-	1.530	0.0716	1.887	0.0887
Glyceric acid	1.558	0.1885	0.591	0.5088
Malic acid	0.590	0.6898	0.970	0.9430
Malonic acid	0.011	0.0941	0.998	0.9960
Succinic acid	1.000	0.1112	0.837	0.6528
Shikimic acid	0.983	0.1482	1.180	0.6661
Pyruvic acid	1.758	0.9149	3.249*	0.0472
Phosphoric acid monomethyl ester	1.590*	0.0466	1.053	0.8588
Phosphoric acid	2.198	0.5017	1.229	0.4907
Isocaproic acid, 2-oxo-	1.000	0.1517	0.882	0.8198
Threonic acid	0.748*	0.0089	0.926	0.8797
*Others metabolites*				
Inositol, myo	0.939*	0.0423	1.140	0.4266
Arabitol	1.199	0.0562	1.160	0.6222
Diethylenglycol	0.935	0.0603	1.916	0.0748
Lyxonic acid-1,4-lactone	0.781*	0.0120	1.299	0.3070
Mannosamine, *N*-acetyl-	0.949	0.0626	1.090	0.7844
Sphingosine	0.961	0.1919	1.307	0.2223
Threonic acid-1,4-lactone	1.071	0.062	1.204	0.4683

The metabolites followed by an asterisk change significantly in their content in *mit-2* with respect to WT samples (*P*<0.05)

Generally, there is mainly an up-regulation of metabolite levels in shoots of *mit-2* plants, compared with the WT. In total, 36 metabolites increased >2-fold (including unknowns; Supplementary Table S5), 22 of them being significantly changed, while five metabolites decreased to half of the WT level but only one of them changed significantly. On the other hand, the metabolite levels in roots are generally down-regulated. Overall, 26 metabolites (including unknowns; Supplementary Table S5) decreased with a <0.5-fold change; 13 of these changes are significant. In contrast, only 13 metabolites increased by >2-fold in the root tissue and only two of these increases were significant.

Specifically, 15 amino acids and related compounds were significantly affected in the mutant plants. In roots, a decreased content (expressed as fold change *mit-2*/WT ratio) of several metabolites, such as alanine, glycine, guanidine, and homoserine, was observed. Additionally, the concentration of other metabolites such as citric acid was significantly decreased in roots of the mutant plants, and a decrease in fructose and sucrose content was also found.

In contrast, in *mit-2* shoots a significant accumulation of eight different metabolites was observed in comparison with WT shoots, These comprised amino acid-related metabolites (ornithine and ethanolamine), organic acids such as fumaric acid, pyruvic acid, and ribonic acid, and some carbohydrates, such as galactose, raffinose, and mannose.

## Discussion

Mitochondria were previously suggested to be involved in Fe sensing and signalling pathways in plant cells (Vigani *et al.*, 2009). The characterization of mutant plants defective in a mitochondrial Fe importer therefore represents a useful tool to investigate the specific involvement of impaired mitochondria in the regulation of nuclear gene expression under Fe deficiency. Indeed, recently the characterization of the rice *oligopeptide 7* mutant (*opt7-1*) showed that such mutant plants display a similar phenotype to that observed in *mit-2* mutant plants: (i) accumulation of Fe in plant tissues and (ii) symptoms of Fe deficiency (Bashir *et al.*, 2015). However, *opt7-1* mutant plants showed different transcriptomic changes compared with what was observed in *mit-2* plants in the present work, highlighting the specific changes with regard to mitochondrial function. To investigate the effect of such mutation on the expression of nuclear genes as well as overall cellular metabolism, a transcriptomic and metabolomic analysis of both root and leaf tissues was performed.

### *mit-2* mutation affects root metabolism by inducing alternative respiratory pathways and related changes in the level of amino acids of the aspartate family

In *mit-2* plants, mitochondrial functionality is affected in the root. This mutation led to a low Fe content in mitochondria with a decreased expression of Fe-containing proteins (i.e Rieske and AOX). At the same time, the NDB1 and NDA1 proteins, belonging to the alternative type II NAD(P)H dehydrogenase (ND-DHs), accumulate in *mit-2* purified mitochondria compared with WT plants. It has been demonstrated that the induction of ND-DH activities is required to bypass a decreased activity of complex I occurring in Fe-deficient Strategy I plants (Higa *et al.*, 2010; Vigani and Zocchi, 2010). Furthermore, the activation of ND-DHs mediates the NAD(P)H oxidation in mitochondria under anaerobic conditions (Igamberdiev and Hill, 2009). However, in roots of *mit-2* plants, an impairment of the glycolytic pathway has been observed, without the induction of transcript related to the fermentative pathway. In Fe-deficient Strategy I plants, mitochondrial impairment was associated with an induction of glycolytic flux as well as fermentative pathways (Vigani *et al.*, 2012).

Together with the mitochondrial alteration, a general slowing down of metabolism has been observed in roots. Indeed, the glycolytic pathway is strongly impaired. Only genes encoding the fructose-2,6-bisphosphatase (F2,6BPase) are strongly up-regulated when compared with WT plants, while other genes belonging to the glycolytic pathway are down-regulated. Accordingly, the contents of glycolysis-related metabolites (glucose, glucose-6-phosphate, and fructose-1-phosphate) were lower in mutant than in WT plants. At the same time, in roots of mutant plants, the down-regulation of genes belonging to secondary metabolism was observed.

**Fig. 5.**
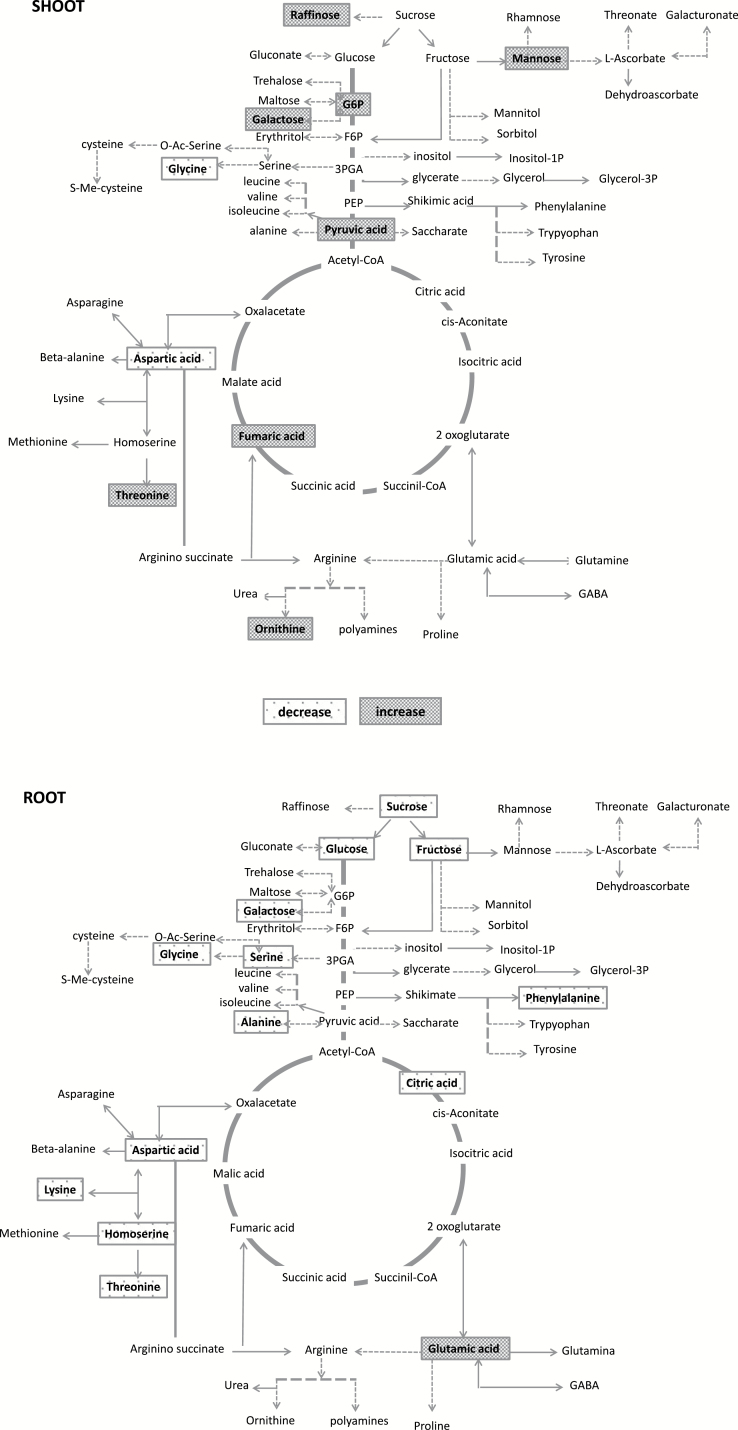
Schematic and synthetic representation of changes of some metabolites in both shoot (upper panel) and root (lower panel) *mit-2* tissues. A significant increase (grey boxes) or decrease (white stippled boxes) in metabolite content in *mit-2* plants compared with the WT is shown.

However, the decreased content of some metabolites does not necessarily imply a metabolic slow down of the respiratory pathway. Interestingly, the significant decrease in citric acid content in roots of *mit-2* plants deserves a particular mention. Indeed citric acid is a Fe(III) chelator and is thought to play a relevant role in xylem Fe transport (Inoue *et al.*, 2004; Yokosho *et al.*, 2009; Rellan-Alvarez *et al.*, 2010). As a higher concentration of Fe was observed in *mit-2* shoots (Bashir *et al.*, 2011c), we suggest that the partial loss of MIT might channel more citrate to chelate Fe and translocate it to the shoot. To enhance citrate synthesis, the TCA cycle should operate at a high rate. Apart from glycolysis, one of the pathways feeding the TCA cycle is the aspartate family pathway (Kirma *et al.*, 2012). Such a pathway synthesizes, through several different metabolic branches, five amino acids (lysine, threonine, methionine, isoleucine, and glycine); all of them can be catabolized in the TCA cycle in order to contribute to cellular energy supply (Kirma *et al.*, 2012). In roots of *mit-2* plants, a decrease in energy supply to the cell by mitochondria, as well as a decrease of glycolysis, occurred (Figs 1, 2); therefore, a need to supply substrates for the TCA cycle by alternative pathways (i.e. the aspartate family pathway) should be expected (Fig. 5). Indeed, in *mit-2* plants, the content of aspartic acid decreased in roots, together with the aspartic acid-related compounds (lysine, homoserine, threonine, and isoleucine).

### *mit-2* mutation affects shoot metabolism by inducing ornithine and RFO accumulation

Bashir *et al.* (2011c) showed that in the shoot *mit-2* mutation affects mitochondrial Fe content and assembly of the Fe–S cluster as well as the aconitase activity which decreased compared with WT plants. Despite the fact that mitochondria were affected in both root and shoot tissues of *mit-2* plants, the metabolic reprogramming in the shoot differs significantly from that observed in roots. Mitochondrial Fe deficiency might have led to a slowdown of glycolysis, as mitochondria may not be able to metabolize pyruvic acid supplied by glycolysis. Accordingly, a clear induction of genes encoding transcripts involved in fermentation together with an increase in glucose-6 phosphate (G6P) and pyruvic acid in the shoot of the mutant could be indicative of a stimulation of fermentative pathways. Despite the fact that *mit-2* plants were chlorotic, the Fe content in the shoot was higher than in WT plants (Bashir *et al.*, 2011c). Therefore, Fe supply to the chloroplast does not seem to be significantly affected by MIT mutation and thereby chloroplasts would be working, and consequently supplying energy to the *mit-2* plants. Moreover, genes belonging to secondary metabolism were generally up-regulated in shoots of mutant plants, which is in agreement with the observed phenylalanine accumulation.

These results are opposite to those observed in the roots and suggest that knocking down *MIT* determines a deficit in the energy supply of the cell, slowing down cellular metabolism. Similar changes were observed at the metabolite level. The content of the majority of metabolites was decreased in roots, while the content increased for only a few of them. In shoots, the response was opposite: most of the metabolites showed increased levels and only a few metabolites decreased. These findings suggest that the shoots can compensate the impaired mitochondrial activity to some extent by chloroplast functionality and photosynthetic activity, and possibly by additional up-regulation of fermentative pathways.

Similar to roots, in shoot of *mit-2* plants the content of aspartic acid decreased. Such an observed decreased in the shoot might be due to the concomitant accumulation of ornithine: indeed, aspartic acid could generate ornithine through the arginino-succinate lyase which converts arginino-succinate to fumaric acid and arginine, which in turn is converted to ornithine (Fig. 5). Ornithine is a non-protein amino acid playing a central role in the polyamine (PA) amino acid biosynthetic pathway (Majumdar *et al.*, 2013). Indeed, it has been suggested that ornithine might be involved in the monitoring and/or signalling pathway for the biosynthesis of metabolites such as proline, putrescine, γ-aminobutyric acid (GABA), and perhaps also arginine (Majumdar *et al.*, 2013). Interestingly, an additional product of such a biosynthetic pathway is nitric oxide (NO), whose role in various developmental and physiological processes in plants is well documented (Tun *et al.*, 2006; Mur *et al.*, 2013; Tanou *et al.*, 2014). The production of NO also increases under abiotic stress conditions (Wimalasekera *et al.*, 2011) such as Fe and oxygen deficiency (Murgia *et al.*, 2002; Graziano and Lamattina, 2007). Therefore, considering the importance of such a pathway, the existence of a mechanism to control cellular ornithine has been proposed (Minocha *et al.*, 2014). However, ornithine can also be synthesized, along with urea, from arginine through the reaction catalysed by arginase which, in plants, localizes in mitochondria (Taylor *et al.*, 2010). A significant accumulation of ornithine occurred in shoots of *mit-2* plants, suggesting that in this mutant, the arginase-dependent pathway would be responsible for the ornithine accumulation. Accordingly, a strong accumulation of fumaric acid occurred in shoots. Fumaric acid is an intermediate of the TCA cycle and its accumulation might be due to TCA cycle impairment. However it could be synthesized from the reaction catalysed by arginino-succinate lyase which converts arginino-succinate to arginine and fumaric acid (Fig. 5). The slight accumulation of arginine, together with the significant accumulation of ornithine occurring at the shoot level in mutant plants suggests that fumaric acid accumulation might derive from such a pathway, although we cannot decipher other possible sources (Fig. 5).

Recently, a strong accumulation of ornithine, together with arginine, in rice embryos germinated under anaerobic condition has been observed, suggesting an enhanced amino acid metabolism involving these compounds under anaerobic conditions. Therefore, we could suggest that *mit-2* mutation induces accumulation of compounds related to arginine metabolism according to what occurs in anaerobic-germinated seeds of rice plants (Taylor *et al.*, 2010). These findings suggest that knocking down *MIT* expression might mimic some hypoxia-induced responses in rice plants. In graminaceous plants, the responses to Fe deficiency and hypoxia are intimately related: Fe deficiency causes physiological hypoxia (Mori *et al.*, 1991), whereas submergence and hypoxia induce enzymes involved in phytosiderophore and ethylene biosynthesis (Suzuki *et al.*, 1998; Sauter *et al.*, 2005). The possible role of O_2_ as a signal molecule for an Fe-responsive gene in rice has recently been suggested (Kobayashi and Nishizawa, 2014). Furthermore, by knocking down the *MIT* gene, a decline in ATP synthesis would be expected as the respiratory chain activity is decreased (compared with WT plants). Interestingly, several genes encoding enzymes of fermentative pathways were up-regulated in shoots of *mit-2* plants, suggesting that such a mutation could indeed simulate a hypoxia-induced response.

Furthermore, changes in the leaf sugar concentration were also observed in *mit-2* plants. Partial loss of MIT leads to the accumulation of some oligosaccharides of the raffinose family (RFO) such as galactose and raffinose. Interestingly, such changes have also been observed in Fe-deficient *Beta vulgaris* plants (Rellan-Alvarez *et al.*, 2010). As raffinose has hydroxyl radical scavenging activity similar to other soluble antioxidants such as glutathione and ascorbic acid, it has been suggested that a strong increase in the relative amounts of RFOs could play a role in the antioxidant defence (Nishizawa *et al.*, 2008; Van den Ende and Valluru, 2009; Rellan-Alvarez *et al.*, 2010). Indeed, the increase in RFO concentration could help to alleviate ROS damage produced under Fe deficiency (Rellan-Alvarez *et al.*, 2010). Furthermore, the increase in RFOs could also act as a long-distance Fe deficiency signal via phloem sap transport. Considering that RFOs accumulate in *mit-2* plants, such an accumulation might depend on the Fe deficiency perception by mitochondria (Fig. 5).

### Conclusion

Here we show global changes in gene expression and metabolite profiles in different metabolic pathways in *mit-2* plants.

In particular, our findings suggest that (i) localized Fe deficiency perceived specifically in mitochondria affects genomic responses in plants; (ii) partial loss of MIT differentially regulates root and shoot transcriptomes; and, in turn (iii) it differentially reprograms the whole metabolism in root and shoot tissues. The metabolites significantly affected in their content in both the root and shoot of *mit-2* plants mainly belong to the aspartate-related pathways (aspartate, lysine, and threonine in roots; aspartate and ornithine in shoots) that are strictly connected to the TCA cycle. It has been suggested that the biological function of the connection between aspartate family pathways and the TCA cycle might have a major role in the physiological response of plants to various abiotic stresses causing energy deprivation (Kirma *et al.*, 2012). Indeed, MIT deficiency led to changes in gene expression and metabolite levels in shoots that resemble hypoxic responses in plants.

Furthermore, the mitochondria–plastid crosstalk occurring in the roots might differentially influence nuclear gene expression with respect to the mitochondria–chloroplast crosstalk occurring in the shoot. Such crosstalk might be responsible for the accumulation of some metabolites (e.g. ornithine, and RFOs) in the leaf which are considered to act as signal molecules under various stress conditions.

In conclusion, this work provides *in planta* evidence that Fe-deficient mitochondria affect the overall cellular metabolism in plants. Whether such changes are due to the impaired activity of Fe–S cluster-containing enzymes in the cell or to an as yet unknown retrograde signalling pathway still remains to be elucidated. Therefore, further work is required to decipher the role of mitochondria in the regulation of Fe homeostasis in plants.

## Supplementary data

Supplementary data are available at *JXB* online.

Figure S1. Changes in the expression of selected genes validated through real time PCR analysis.

Figure S2. Overview of transcriptomic changes in roots of the *mit-2* mutant as indicated by microarray analysis and visualized with Mapman 3.6.0RC1 (values expressed as log_2_ *mit-2*/WT ratio). 

Figure S3. Overview of transcriptomic changes in shoots of the *mit-2* mutant as indicated by microarray analysis and visualized with Mapman 3.6.0RC1 (values expressed as log_2_ *mit-2*/WT ratio). 

Figure S4. HLC tree of metabolic profiles of root and shoot rice tissues (five independent replicates).

Table S1. Genes significantly up-regulated in roots.

Table S2. Genes significantly down-regulated in roots.

Table S3. Genes significantly up-regulated in shoots.

Table S4. Genes significantly down-regulated in shoots.

Table S5. List of known and unknown metabolites determined by GS-TOF-MS analysis in root and shoot tissues.

Supplementary Data
