# Supplementary material for: Knocking down mitochondrial iron transporter (MIT) reprograms primary and secondary metabolism in rice plants
Source: J Exp Bot. 2015 Dec 17;67(5):1357–68. doi: 10.1093/jxb/erv531 (PMC4762380; doi:10.1093/jxb/erv531)
Supplement: Supplementary Data [file supp_67_5_1357__index.html]

Knocking down mitochondrial iron transporter (MIT) reprograms primary and secondary metabolism in rice plants — Knocking down mitochondrial iron transporter (MIT) reprograms primary and secondary metabolism in rice plants — Supplementary Data 

# Knocking down mitochondrial iron transporter (MIT) reprograms primary and secondary metabolism in rice plants

## Supplementary Data

Data files

- Supplementary\_Figure\_S1\_S4.pdf - Supplementary Data
- supplementary\_table\_S1.xls - Supplementary Data
- Supplementary\_Table\_S2.xlsx - Supplementary Data
- Supplementary\_Table\_S3.xlsx - Supplementary Data
- supplementary\_table\_S4.xlsx - Supplementary Data
- supplementary\_table\_S5.xlsx - Supplementary Data
