# Supplementary material for: Knocking down mitochondrial iron transporter (MIT) reprograms primary and secondary metabolism in rice plants
Source: J Exp Bot. 2015 Dec 17;67(5):1357–68. doi: 10.1093/jxb/erv531 (PMC4762380; doi:10.1093/jxb/erv531)
Supplement: Supplementary Data [file supp_erv531_Supplementary_Figure_S1_S4.pdf]

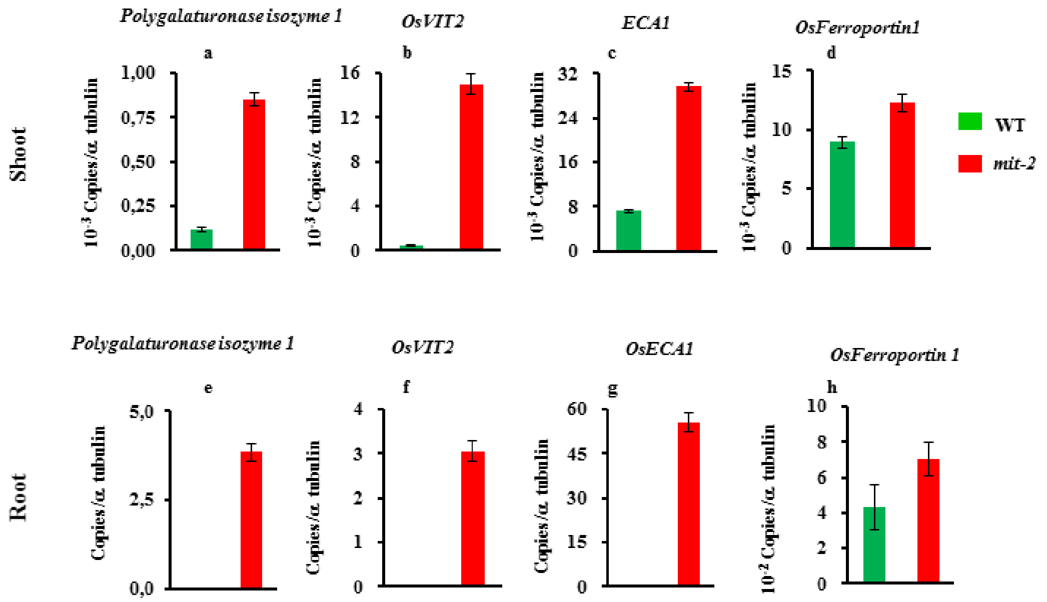

Figure-S1: Changes in the expression of selected genes validated through real time PCR analysis.

a-d Shoot, e-h: root, a, e: *Polygalaturonase isozyme 1 (Os10g0409400)*, b, f: *OsVIT2* (*Os09g0396900*), c, g: *OsECA1* (*Os03g0296600*), d, h: *OsFerroportin 1* (*Os06g0560000*).

Similar to microarray analysis samples from three biological replications were pooled together to make one replication before RNA extraction. Real time PCR analysis were performed with two biological and two technical replications. *α-tubulin* was used for data normalization. Primers used were forward 5`-GATGACATTGTGGTTCGGAGTA-3` and reverse 5`-GATCTCGGCCTCATAAACTCTG-3` for *Polygalaturonase isozyme 1*, forward 5`-GTTTGAGTTGGGACTGGAGAAG-3` and reverse 5`- CCTTGACATAGCCGAAGAAGAG-3` for *OsVIT2*, forward 5`-GGGAGATCCTCCTCTTCTTCAT-3` and reverse 5`- GCAGTAGCCCTTGAGCATGT-3` for *OsECA1*, forward 5`- GATGTTCGACCTAGCCGTGAT-3` and reverse 5`- AGGTCACGAGAAAGAAGGACAG-3` for *OsFerroportin 1*. Forward and reverse primers for *α-tubulin* were 5′-TCTTCCACCCTGAGCAGCTC-3′ and 5′-AACCTTGGAGACCAGTGCAG-3′ respectively.

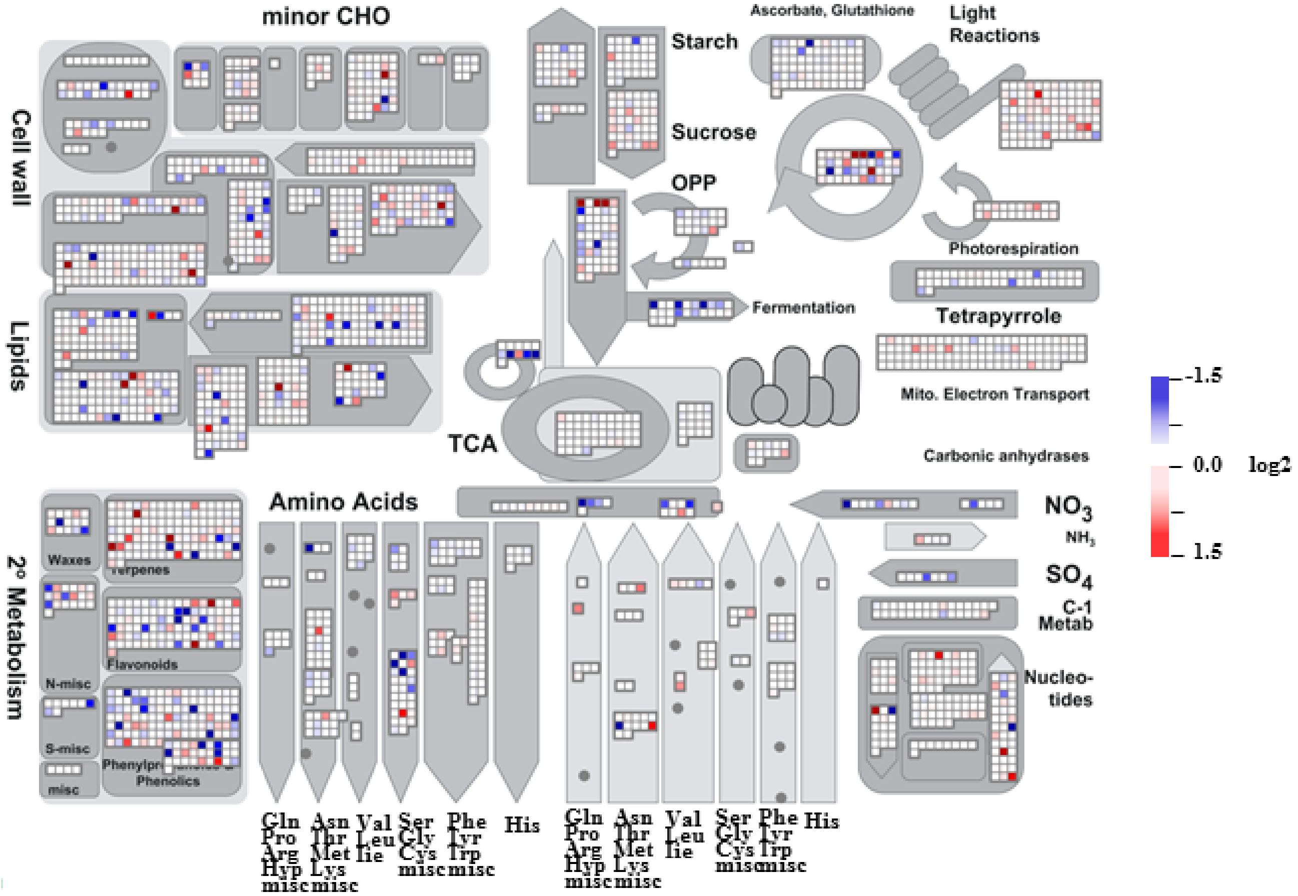

Figure-S2. Overview of transcriptomic changes in roots of *mit-2* mutant as indicated by microarray analysis and visualized with Mapman 3.5.1R2 (values expressed as log2 *mit-2*/WT ratio).
Various components of primary and secondary metabolism including CHO metabolism, cell wall synthesis, lipid metabolism, Calvin cycle changed.

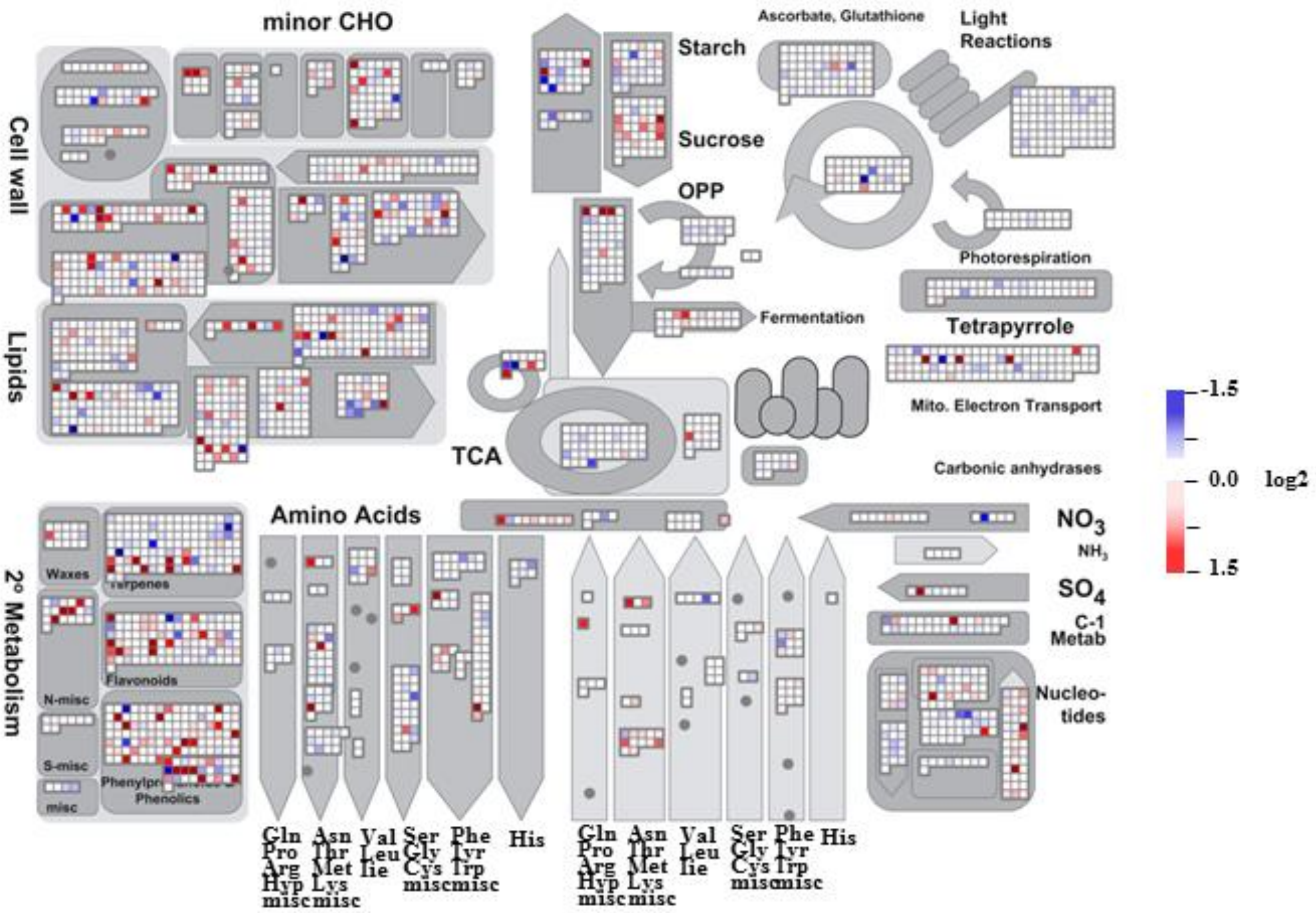

Figure-S3. Overview of transcriptomic changes in shoots of *mit-2* mutant as indicated by microarray analysis and visualized with Mapman 3.5.1R2 (values expressed as log2 *mit-2*/WT ratio). Various components of primary and secondary metabolism including CHO metabolism, cell wall synthesis, lipid metabolism, Calvin cycle changed significanlty.

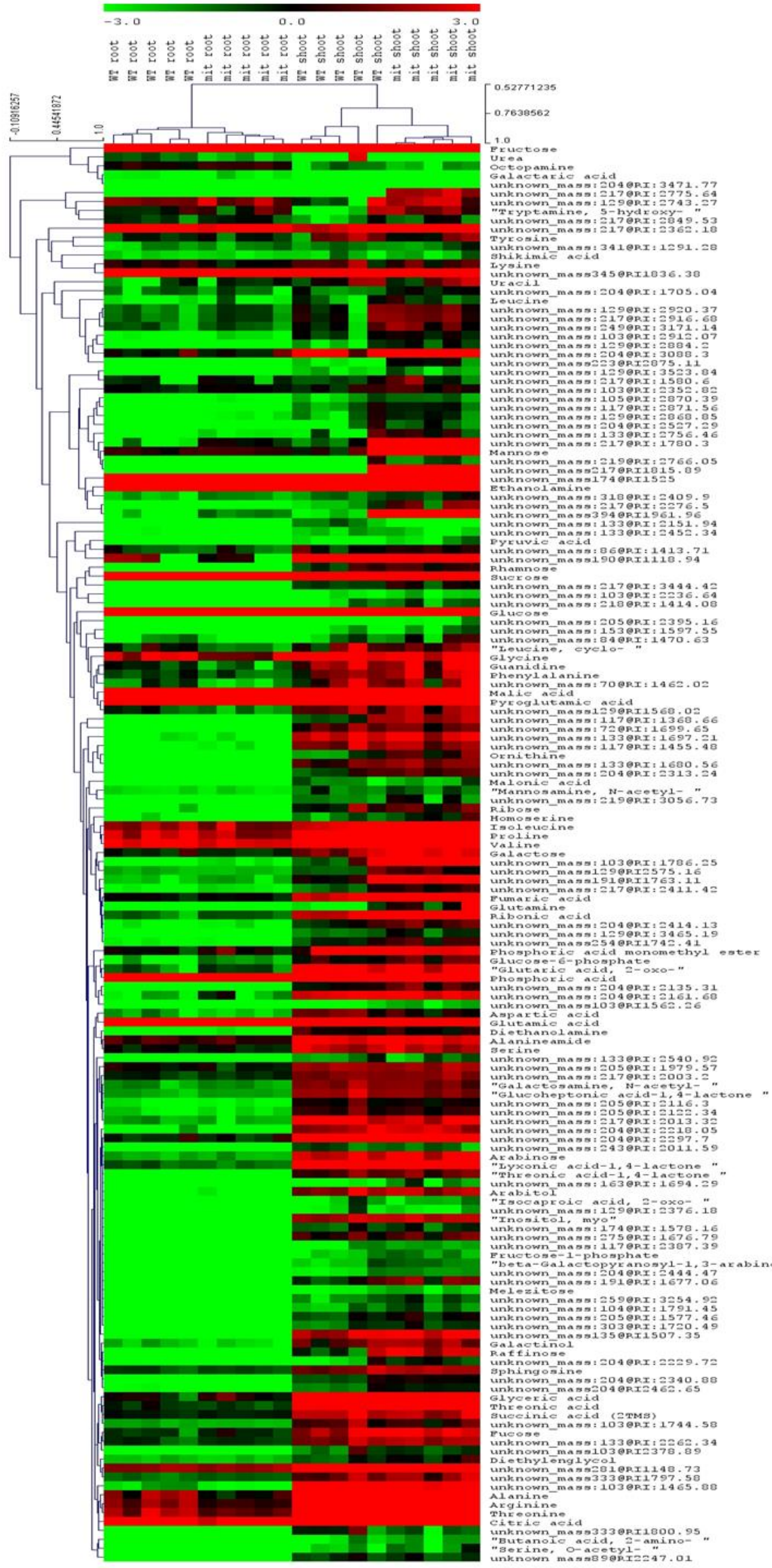

-3.0
0.0
3.0
WT root
WT root
WT root
WT root
WT root
mit root
mit root
mit root
mit root
mit root
WT shoot
WT shoot
WT shoot
WT shoot
WT shoot
mit shoot
mit shoot
mit shoot
mit shoot
mit shoot
0.52771235
0.7638562
1.0
-0.10916257
0.44541872
1.0
Fructose
Urea
Octopamine
Galactaric acid
unknown_mass:204@RI:3471.77
unknown_mass:217@RI:2775.64
unknown_mass:129@RI:2743.27
"Tryptamine, 5-hydroxy- "
unknown_mass:217@RI:2849.53
unknown_mass:217@RI:2362.18
Tyrosine
unknown_mass:341@RI:1291.28
Shikimic acid
Lysine
unknown_mass345@RI1836.38
Uracil
unknown_mass:204@RI:1705.04
Leucine
unknown_mass:129@RI:2920.37
unknown_mass:217@RI:2916.68
unknown_mass:249@RI:3171.14
unknown_mass:103@RI:2912.07
unknown_mass:129@RI:2884.2
unknown_mass:204@RI:3088.3
unknown_mass223@RI2875.11
unknown_mass:129@RI:3523.84
unknown_mass:217@RI:1580.6
unknown_mass:103@RI:2352.82
unknown_mass:105@RI:2870.39
unknown_mass:117@RI:2871.56
unknown_mass:129@RI:2868.85
unknown_mass:204@RI:2527.29
unknown_mass:133@RI:2756.46
unknown_mass:217@RI:1780.3
Mannose
unknown_mass:219@RI:2766.05
unknown_mass217@RI1815.89
unknown_mass174@RI1525
Ethanolamine
unknown_mass:318@RI:2409.9
unknown_mass:217@RI:2276.5
unknown_mass394@RI1961.96
unknown_mass:133@RI:2151.94
unknown_mass:133@RI:2452.34
Pyruvic acid
unknown_mass:86@RI:1413.71
unknown_mass190@RI1118.94
Rhamnose
Sucrose
unknown_mass:217@RI:3444.42
unknown_mass:103@RI:2236.64
unknown_mass:218@RI:1414.08
Glucose
unknown_mass:205@RI:2395.16
unknown_mass:153@RI:1597.55
unknown_mass:84@RI:1470.63
"Leucine, cyclo- "
Glycine
Guanidine
Phenylalanine
unknown_mass:70@RI:1462.02
Malic acid
Pyroglutamic acid
unknown_mass129@RI1568.02
unknown_mass:117@RI:1368.66
unknown_mass:72@RI:1699.65
unknown_mass:133@RI:1697.21
unknown_mass:117@RI:1455.48
Ornithine
unknown_mass:133@RI:1680.56
unknown_mass:204@RI:2313.24
Malonic acid
"Mannosamine, N-acetyl- "
unknown_mass:219@RI:3056.73
Ribose
Homoserine
Isoleucine
Proline
Valine
Galactose
unknown_mass:103@RI:1786.25
unknown_mass129@RI2575.16
unknown_mass191@RI1763.11
unknown_mass:217@RI:2411.42
Fumaric acid
Glutamine
Ribonic acid
unknown_mass:204@RI:2414.13
unknown_mass:129@RI:3465.19
unknown_mass254@RI1742.41
Phosphoric acid monomethyl ester
Glucose-6-phosphate
"Glutaric acid, 2-oxo-"
Phosphoric acid
unknown_mass:204@RI:2135.31
unknown_mass:204@RI:2161.68
unknown_mass103@RI1562.26
Aspartic acid
Glutamic acid
Diethanolamine
Alanineamide
Serine
unknown_mass:133@RI:2540.92
unknown_mass:205@RI:1979.57
unknown_mass:217@RI:2003.2
"Galactosamine, N-acetyl- "
"Glucoheptonic acid-1,4-lactone "
unknown_mass:205@RI:2116.3
unknown_mass:205@RI:2122.34
unknown_mass:217@RI:2013.32
unknown_mass:204@RI:2218.05
unknown_mass:204@RI:2297.7
unknown_mass:243@RI:2011.59
Arabinose
"Lyxonic acid-1,4-lactone "
"Threonic acid-1,4-lactone "
unknown_mass:163@RI:1694.29
Arabitol
"Isocaproic acid, 2-oxo- "
unknown_mass:129@RI:2376.18
"Inositol, myo"
unknown_mass:174@RI:1578.16
unknown_mass:275@RI:1676.79
unknown_mass:117@RI:2387.39
Fructose-1-phosphate
"beta-Galactopyranosyl-1,3-arabinose, D-
unknown_mass:204@RI:2444.47
unknown_mass:191@RI:1677.06
Melezitose
unknown_mass:259@RI:3254.92
unknown_mass:104@RI:1791.45
unknown_mass:205@RI:1577.46
unknown_mass:303@RI:1720.49
unknown_mass135@RI1507.35
Galactinol
Raffinose
unknown_mass:204@RI:2229.72
Sphingosine
unknown_mass:204@RI:2340.88
unknown_mass204@RI2462.65
Glyceric acid
Threonic acid
Succinic acid (2TMS)
unknown_mass:103@RI:1744.58
Fucose
unknown_mass:133@RI:2262.34
unknown_mass103@RI2378.89
Diethylenglycol
unknown_mass281@RI1148.73
unknown_mass333@RI1797.58
unknown_mass:103@RI:1465.88
Alanine
Arginine
Threonine
Citric acid
unknown_mass333@RI1800.95
"Butanoic acid, 2-amino- "
"Serine, O-acetyl- "
unknown mass89@RI2247.01
